# Clinical implications of *Mycobacterium chimaera* detection in thermoregulatory devices used for extracorporeal membrane oxygenation (ECMO), Germany, 2015 to 2016

**DOI:** 10.2807/1560-7917.ES.2016.21.46.30398

**Published:** 2016-11-17

**Authors:** Franziska C. Trudzinski, Uwe Schlotthauer, Annegret Kamp, Kai Hennemann, Ralf M. Muellenbach, Udo Reischl, Barbara Gärtner, Heinrike Wilkens, Robert Bals, Mathias Herrmann, Philipp M. Lepper, Sören L. Becker

**Affiliations:** 1 Department of Medicine V – Pneumology, Allergology and Critical Care Medicine, ECLS Center Saar, Saarland University, Homburg/Saar, Germany; 2 Institute of Medical Microbiology and Hygiene, Saarland University, Homburg/Saar, Germany; 3 Department of Thoracic and Cardiovascular Surgery, Saarland University, Homburg/Saar, Germany; 4 Department of Anaesthesiology and Critical Care, Campus Kassel of the University Hospital of Southampton, Kassel, Germany; 5 Institute of Clinical Microbiology and Hygiene, University Hospital Regensburg, University of Regensburg, Regensburg, Germany; 6 Faculty of Medicine, University of Münster, Münster, Germany; 7 Swiss Tropical and Public Health Institute, Basel, Switzerland; 8 University of Basel, Basel, Switzerland

**Keywords:** *Mycobacterium chimaera*, atypical mycobacteria, infection, diagnosis, heater-cooler unit, extracorporeal membrane oxygenation (ECMO)

## Abstract

*Mycobacterium chimaera*, a non-tuberculous mycobacterium, was recently identified as causative agent of deep-seated infections in patients who had previously undergone open-chest cardiac surgery. Outbreak investigations suggested an aerosol-borne pathogen transmission originating from water contained in heater-cooler units (HCUs) used during cardiac surgery. Similar thermoregulatory devices are used for extracorporeal membrane oxygenation (ECMO) and *M. chimaera* might also be detectable in ECMO treatment settings. We performed a prospective microbiological study investigating the occurrence of *M. chimaera* in water from ECMO systems and in environmental samples, and a retrospective clinical review of possible ECMO-related mycobacterial infections among patients in a pneumological intensive care unit. We detected *M. chimaera* in 9 of 18 water samples from 10 different thermoregulatory ECMO devices; no mycobacteria were found in the nine room air samples and other environmental samples. Among 118 ECMO patients, 76 had bronchial specimens analysed for mycobacteria and *M. chimaera* was found in three individuals without signs of mycobacterial infection at the time of sampling. We conclude that *M. chimaera* can be detected in water samples from ECMO-associated thermoregulatory devices and might potentially pose patients at risk of infection. Further research is warranted to elucidate the clinical significance of *M. chimaera* in ECMO treatment settings.

## Introduction

*Mycobacterium chimaera* is a slowly growing atypical mycobacterium that is closely related to the more commonly encountered species *M. avium* and *M. intracellulare* [1]. The potential of *M. chimaera* to cause clinical disease was previously considered to be low [2,3], however, a multi-country outbreak of severe infections due to *M. chimaera* was recently described in patients who had undergone open-chest cardiac surgery [4]. Indeed, *M. chimaera* was identified as the causative agent of deep-seated infections such as endocarditis and vertebral osteomyelitis in patients from different European countries (e.g. Germany, the Netherlands, Switzerland) [5,6] and from North America [7]. Interestingly, these infections occurred up to 5 years after the patients had been exposed to cardiothoracic surgical procedures, during which heater-cooler units (HCUs) were used. 

Atypical mycobacteria can be detected in household water [8] and water-containing medical devices [9-11], and it had thus been suggested that the HCUs, which use water for thermoregulation during cardioplegia, might constitute the common source of the recent outbreak [12]. Indeed, an air-borne transmission of M. *chimaera* in the operating room was confirmed [13] and a report by Haller et al. provided evidence that at least some of the HCUs might already have been contaminated at the manufacturing site [14]. Preventive measures to reduce the risk of transmission during cardiac surgery are now being implemented worldwide. Meanwhile, it remains to be elucidated whether other water-containing medical devices might also pose patients at risk of acquiring infections due to *M. chimaera*.

Veno-venous extracorporeal membrane oxygenation (ECMO) is an established treatment for patients with severe acute respiratory distress syndrome (ARDS) [15,16]. Additionally, veno-arterial circuit configurations offer a prolonged circulatory support, which is comparable to the short-term support provided by cardiopulmonary bypass (CPB) during cardiac surgery [17-20]. All extracorporeal circuits (ECC) consist of a tubing system, which is connected to a roller or centrifugal pump to maintain the active blood transport and guides the flow through the membrane oxygenator. Control units are used to adjust the blood flow between 2 and 7 L per minute. Thermoregulatory devices, heater units (HUs) or HCUs are engaged to adjust the blood temperature within the ECC [21]. All commercially available thermoregulatory systems run in analogy to those used in the operating room with circulating water. Hence, it may be hypothesised that ECMO treatment might also constitute a risk for transmission of water-borne pathogens. While patients treated with ECMO for respiratory failure have smaller potential entry sites for pathogens than those undergoing open-chest heart surgery, they are nevertheless critically ill and highly immunocompromised, and are thus susceptible to opportunistic infections. Additionally, as patients may be subjected to ECMO treatment for a prolonged duration of up to several months [22,23], there is a need to assess their potential exposure to water-borne pathogens such as *M. chimaera* in thermoregulatory devices used for ECMO.

Here, we present an in-depth assessment on the occurrence of *M. chimaera* in an ECMO centre in Germany, with a particular focus on potential mycobacterial transmission pathways and the clinical significance arising from our findings. Our investigation comprises two specific parts, i.e. (i) a prospective microbiological sampling of water from ECMO devices and from the environment for *M. chimaera* (August 2015–August 2016); and (ii) a retrospective patient chart analysis to identify potentially exposed individuals with positive *M. chimaera* culture results and previous ECMO treatment (April 2010–June 2016).

## Methods

### Study site and study procedures

The current study was carried out at the pneumological intensive care unit (ICU) at Saarland University Medical Center in Homburg, southwest Germany. This medical centre is a supra-regional ECMO centre and provides approximately 20 lung transplantations per year. Microbiological investigations pertaining to the presence of atypical mycobacteria in HCUs used during cardiac surgery were initiated in March 2015, and the prospective sampling of water from devices used for ECMO treatment was subsequently started in August 2015. Prompted by these microbiological investigations, a retrospective patient chart review of individuals treated with ECMO at our centre in the preceding 6 years was initiated in mid-2016 to further assess the significance of *M. chimaera* in this specific setting.

### Characteristics of extracorporeal circuits and thermoregulatory devices

**Figure 1:**
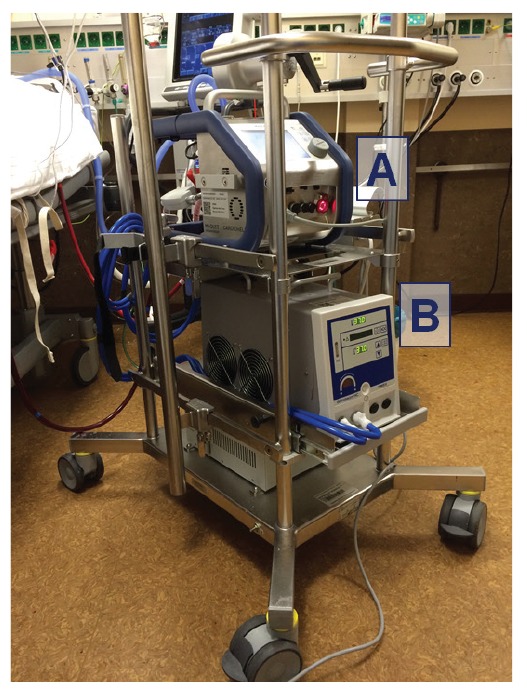
The functional set-up of an ECMO treatment unit, consisting of (A) an ECMO system; and (B) a thermoregulatory device at a medical intensive care unit, Homburg/Saar, Germany

During the study period, veno-venous ECMO cannulation was performed using the femoral (draining) and jugular (return) veins as main cannula entry sites. As a standard, we used 23 F draining cannulae at a length of 38 or 55 cm, as appropriate, and 19 F returning cannulae (Maquet Holding B.V. and Co. KG, Rastatt, Germany) with a heparin coating. Some patients underwent single stage cannulation using a bicaval double-lumen cannula (27 F or 31 F Avalon Elite, Avalon Laboratories; Rancho Dominguez, United States of America). Standard oxygenators were 7.0L-HLS or Quadrox-I with a ROTAFLOW Centrifugal Pump RF 32 primed with physiological saline solution used on the Maquet CardioHelp or ROTAFLOW platform. Different thermoregulatory devices were used according to individual requirements; heater units such as ‘Heater Unit HU 35’ (Maquet) or HCUs such as ‘Deltastream HC’ (Medos Medizintechnik AG; Stolberg, Germany) and ‘NovaTherm’ (Novalung GmbH; Heilbronn, Germany). The functional set-up of an ECMO system with a heater unit is shown in Figure 1.

ECMO: extracorporeal membrane oxygenation.

**Figure 2:**
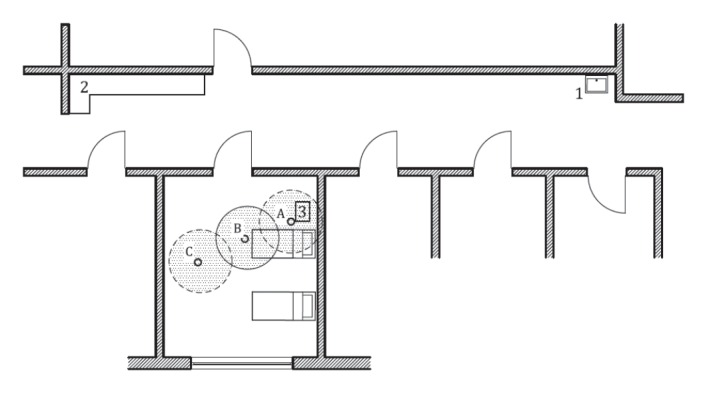
Ground plan of the intensive care unit, Homburg/Saar, Germany

All thermoregulatory devices were temporarily leased from the manufacturers and filled with filtered tap water (Aquasafe filter AQ31F1S, PALL Corporation; Dreieich, Germany; filter width: 0.2 μm). The priming of all circuits and corresponding thermoregulatory devices was performed within a central priming area and the devices were then placed bedside while being used. A schematic diagram of these operational areas is shown in Figure 2.

ECMO: extracorporeal membrane oxygenation.

The figure schematically shows the floor with the individual patient rooms where sampling took place. Water for thermoregulatory devices was taken from a sink (1) on the ward. The sink is equipped with a Pall Aquasafe filter to avoid microbial contamination. The filter is changed every 30 days, as recommended by the manufacturer. ECMO units are primed with sterile saline solution in the priming area (2). The ECMO machine and the thermoregulatory device are installed at the bedside (3) of the patient. Water samples were taken from the tap (1) and from the thermoregulatory devices. Air sampling took place (A) next to the ECMO device; (B) next to the patient; and (C) at 2–3 m distance from the patient and the ECMO device, but in the same room.

### Processing and microbiological analysis of water from ECMO devices, tap water and environmental samples

For investigation of atypical mycobacteria, 100–250 mL of water were collected from the water tanks of thermoregulatory devices used for ECMO treatment, and were processed according to a protocol issued by the European Centre for Disease Prevention and Control (ECDC) [24]. In brief, water samples were concentrated by centrifugation and subsequently decontaminated using N-acetyl-L-cysteine sodium hydroxide (NALC-NaOH). Additional examinations were carried out on filtered tap water (filter width 0.2 µm) that was used to fill the tanks of the ECMO devices. Of note, the tap water used is also regularly checked for compliance with the German drinking water directive [25]. Microscopy using auramine staining was carried out on all samples, and water samples were plated on two different media, i.e. (i) 7H11 Middlebrook agar; and (ii) Löwenstein-Jensen agar. Additionally, cultures in liquid media were also performed and water samples were inoculated into the MGIT 960 system. All culture media were obtained from Becton Dickinson (Heidelberg, Germany) and were incubated for up to 8 weeks.

Environmental room air sampling was carried out in patient rooms during ECMO treatment while thermoregulatory devices, which had previously tested positive for *M. chimaera*, were running. For each sampling, 100–200 L of room air was collected using the MBASS 30 microbiological air sampling system (Umweltanalytik Holbach GmbH; Holbach, Germany) and conducted over selective 7H10 Middlebrook agar plates during one minute. During each sampling, air specimens were taken at three different locations, i.e. (i) next to the ECMO device; (ii) next to the patient; and (iii) in 2–3 m distance from the patient and the ECMO device, but in the same room. Additionally, swabs (eSwab, Copan Diagnostics; Brescia, Italy) were taken once from the surface and connecting tubes of selected ECMO thermoregulatory devices, and were subsequently analysed for the presence of mycobacteria. All agar plates were examined twice weekly during eight weeks for signs of mycobacterial growth. Suspicious colonies were identified to the species level using a commercially available molecular typing system (GenoType NTM-DR, Hain Lifescience; Nehren, Germany). Additionally, a subsample of positive specimens was sent to a reference centre for molecular diagnostics at the University Hospital Regensburg, Germany, where the species identification of *M. chimaera* was confirmed by partial sequencing of the 16S, ITS and *rpoB* gene sequences.

### Retrospective patient analysis and microbiological work-up of patient samples

Using an electronic database, we retrospectively identified all patients undergoing ECMO treatment (excluding extracorporeal CO_2_ removal; ECCO_2_R) at the pneumological ICU at Saarland University Medical Center between April 2010 and June 2016. 

Respiratory samples were taken if clinical signs and symptoms of respiratory infection were present and/or whenever a potential infection was clinically suspected. Bronchial specimens obtained during or after ECMO treatment were reviewed both clinically and microbiologically for findings suggestive of mycobacterial infection. 

Due to the retrospective nature of the analysis, no specific protocol was implemented before the start of the study for the microbiological work-up of patient samples. Standard diagnostic procedures were followed and bronchial aspirates and bronchoalveolar lavage specimens of patients undergoing ECMO treatment were immediately sent to the microbiology laboratory using a pneumatic transport system. Upon receipt at the laboratory, samples were decontaminated using NALC-NaOH and mycobacterial examinations were carried out as follows: (i) microscopy using Kinyoun or auramine staining; (ii) MGIT 960 liquid media culture; and (iii) culture on solid agar media (Löwenstein-Jensen agar; Stonebrink agar). All patient samples were incubated for up to 8 weeks and mycobacteria were identified as described above. 

Species identification of *M. chimaera* had not uniformly been performed between 2010 and 2015, thus all isolates that had previously been identified as either *M. intracellulare* or *M. avium* were re-cultured from a biobank and subjected to molecular genetic testing for unambiguous species identification.

## Results

### Detection of *Mycobacterium chimaera* in water and air samples

Between August 2015 and August 2016, a total of 18 water samples originating from 10 different thermoregulatory devices used for ECMO treatment were subjected to microbiological analyses. *M. chimaera* was detected in nine specimens i.e. half of all examined water samples. Of the ten analysed thermoregulatory devices, water obtained from seven tested positive in at least one sample. In five water samples, mycobacteria were visible by microscopy, which suggests presence of a relatively high number of mycobacteria. The liquid medium culture (MGIT) was earliest to give positive results in all cases, after approximately 2 weeks of incubation (10–16 days). In one water sample which tested positive for *M. chimaera*, a co-colonisation with *M. gordonae* was observed. Details on the microbiological test results are given in Table 1.

**Table 1:** Mycobacterial testing characteristics of water samples from thermoregulatory devices used for ECMO treatment at a pneumological intensive care unit, Germany, 2015–2016

Patient (n = 18)	Thermoregulatory device (n = 10)	Device model (Manufacturer)	Sampling date	Microscopy	Culture	Species identification
**1**	1	Deltastream HC (Medos)	August 2015	**Positive ( + + + )**	**Positive**	*M. chimaera*
**2**	2	Deltastream HC (Medos)	August 2015	Negative	**Positive**	*M. chimaera*
**3**	3	HU35 (Maquet)	December 2015	Negative	Negative	–
**4**	4	Deltastream HC (Medos)	January 2016	Negative	Negative	–
**5**	5	Deltastream HC (Medos)	January 2016	**Positive ( + )**	**Positive**	*M. chimaera*
**6**	4	Deltastream HC (Medos)	January 2016	Negative	**Positive**	*M. chimaera*
**7**	6	Deltastream HC (Medos)	January 2016	Negative	Negative	–
**8**	7	HU35 (Maquet)	January 2016	Negative	Negative	–
**9**	4	Deltastream HC (Medos)	March 2016	**Positive ( + )**	**Positive**	*M. chimaera* and *M. gordonae*
**10**	8	NovaTherm (NovaLung)	March 2016	Negative	Negative	–
**11**	9	Deltastream HC (Medos)	March 2016	Negative	Negative	–
**12**	4	Deltastream HC (Medos)	March 2016	**Positive ( + + + )**	**Positive**	*M. chimaera*
**13**	6	Deltastream HC (Medos)	March 2016	Negative	Negative	–
**14**	9	Deltastream HC (Medos)	April 2016	Negative	Negative	–
**15**	6	Deltastream HC (Medos)	April 2016	Negative	**Positive**	*M. chimaera*
**16**	8	NovaTherm (Novalung)	April 2016	Negative	**Positive**	*M. chimaera*
**17**	9	Deltastream HC (Medos)	August 2016	**Positive ( + + )**	**Positive**	*M. chimaera*
**18**	10	Deltastream HC (Medos)	August 2016	Negative	Negative	–

ECMO: extracorporeal membrane oxygenation; M: *Mycobacterium*.

For microscopy of auramine-stained slides, the following semi-quantitative grading scheme was adopted: (i) negative (no mycobacteria seen on the microscope slide); (ii) + (up to 50 mycobacteria seen per 100 observation fields); (iii) ++ (5-50 mycobacteria seen per 10 observation fields); and (iv) +++ (≥5 mycobacteria seen per observation field).

Filtered tap water, which was commonly used to fill the thermoregulatory devices for ECMO treatment, was subjected to microbiological examinations at three different time points (several weeks apart), but neither bacterial nor mycobacterial pathogens were detected.

When analysing nine room air samples from the pneumological ICU, no atypical mycobacteria and no non-fermentative Gram-negative rods were detected during 8 weeks of incubation. Of note, some specimens contained low quantities of environmental Gram-positive (e.g. *Arthrobacter* spp., *Bacillus subtilis*, *Corynebacterium amycolatum* and *Micrococcus luteus*) and Gram-negative bacteria (e.g. *Moraxella osloensis*). Environmental moulds (e.g. *Penicillium citrinum*) were also found.

A series of 12 swabs taken from different surfaces and connecting tubes of two running ECMO thermoregulatory devices remained uniformly negative for *M. chimaera*.

### Occurrence of *Mycobacterium chimaera* in patients treated with ECMO

We reviewed the electronic charts of all 118 patients who had received ECMO support between April 2010 and June 2016. Bronchial specimens (bronchial aspirates and/or bronchoalveolar lavage samples) from 79 patients (67.0%) were analysed for the presence of mycobacteria during or after ECMO therapy. All patients received respiratory ECMO support either due to severe ARDS or as a temporary ‘bridging procedure’ to planned lung transplantation. A total of 32 of 79 (40.5%) patients were male and the mean age was 46.8 years (standard deviation (SD): 16.7 years). The mean duration of ECMO treatment was 20.2 days (SD: 46.6 days), and 58.3% of the analysed patients survived to discharge.

Mycobacteria were observed upon microscopy (auramine staining) in bronchial specimens from one individual among the 79 patients. In three cases, mycobacterial cultures were bacterially contaminated and could not be analysed, thus leading to a final cohort of 76 patients with mycobacterial culture results after the onset of ECMO treatment. Cultures for mycobacteria were positive in four patients and the mycobacterial species were identified as *M. chimaera* in three and *M. malmoense* in one of them. The three cases of *M. chimaera* were critically reviewed to investigate the possible clinical significance of this finding. Brief descriptions on the patient characteristics are given below and in Table 2.

**Table 2:** Characteristics and clinical course of patients diagnosed with *Mycobacterium chimaera* in respiratory specimens while treated with ECMO at a pneumological intensive care unit, Germany, 2010–2016 (n=3)

Patient number	Sex	Age (years)	Underlying disease and operative intervention	Indication for ECMO	Time of ECMO treatment (days)	Risk factor for *M. chimaera*	Days from ECMO treatment onset to sampling for *M. chimaera*	Clinical course
1	Male	Mid 70s	CTEPH, PEA and CABG	ARDS	48	Previous open-chest cardiac surgery	5	Died on ECMO (cardiogenic shock)
2	Male	End 20s	AML, allogenic SCT	GVHD	113	None	6	Died on ECMO (septic shock)
3	Female	Early 30s	CF, LTx, CLAD	ARDS	40	Previous open-chest cardiac surgery	205	Survived (re-LTx)

AML: acute myeloid leukaemia; ARDS: acute respiratory distress syndrome; CABG: coronary artery bypass grafting; CF: cystic fibrosis; CLAD: chronic lung allograft dysfunction; CTEPH: chronic thromboembolic pulmonary hypertension; ECMO: extracorporeal membrane oxygenation; GVHD: graft vs. host disease; LTx: lung transplantation; PEA: pulmonary endarterectomy; re-LTx: lung retransplantation; SCT: stem cell transplantation.

#### Patient 1

In 2010, a man in his mid-70s developed severe acute respiratory distress syndrome (ARDS) and received ECMO therapy after pulmonary endarterectomy and coronary artery bypass grafting had been performed as treatment for chronic thromboembolic pulmonary hypertension and coronary heart disease. Eight days before ECMO initiation, the patient was screened for mycobacteria and was negative. On day 5 with ECMO support, a bronchial specimen was obtained that yielded *M. chimaera*. The patient died in cardiogenic shock after 48 days of ECMO treatment.

#### Patient 2

In 2013, a man in his late 20s underwent allogenic stem cell transplantation for acute myeloid leukaemia, which he developed after treatment of Hodgkin’s lymphoma with thymic infiltration. The patient developed graft vs. host disease with pulmonary involvement. Due to progressive respiratory failure, he was treated with ECMO with the intention to bridge the time to lung transplantation. After 6 days with ECMO, a bronchial aspirate was sent to the laboratory and *M. chimaera* was found once, but not in follow-up examinations 2 and 4 weeks later. Some weeks later, the patient was temporarily treated with clarithromycin, rifampicin, ethambutol and moxifloxacin for a clinically suspected mycobacterial infection. The patient died 113 days after initiation of ECMO therapy in septic shock with bacteraemia due to *Enterococcus faecium*.

#### Patient 3

A woman in her 30s with cystic fibrosis developed a restrictive chronic allograft dysfunction with consecutive lung failure after previous lung transplantation. Hence, she was treated with ECMO in October 2014 and re-transplanted after 40 days with extracorporeal support. She had multiple bronchial aspirates being sampled for the presence of mycobacteria before ECMO (last sampling 11 days earlier) that were always negative. Two hundred five days after ECMO initiation, *M. chimaera* was detected in a bronchial aspirate. The patient is still alive (> 650 days) and her clinical condition is good.

## Discussion

In the present single-centre study, *M. chimaera* was detected in a considerable amount of water samples taken from different thermoregulatory devices of two different providers during ECMO treatment. Indeed, half of all analysed specimens grew *M. chimaera*, whereas no mycobacteria were found in room air samples and swabs from ECMO system surfaces. *M. chimaera* was also detected in three ECMO patients in a retrospective analysis over 6 years, but the transmission pathways as well as the clinical relevance of the findings remain uncertain.

*M. chimaera* was recently described as the causative agent in a multi-country outbreak of severe invasive infections, and pathogen transmission likely occurred through contaminated HCUs used during cardiac surgery [4,5,7,14]. By acknowledging the aetiological role of *M. chimaera* in this outbreak, its clinical relevance had to be reconsidered because previous studies had described *M. chimaera* to be of rather low pathogenicity [26]. Indeed, an analysis of 97 culture isolates from German patients detected a clinical relevance in merely 3.3% of all samples [2], and there is only a limited number of case reports providing evidence of infections due to *M. chimaera* in immunocompromised patients, such as those with severe anorexia nervosa [27], chronic obstructive pulmonary disease [28] and cystic fibrosis [29]. The patients on ECMO treatment described in our report were also immunocompromised and might thus have been at risk of clinical *M. chimaera* infection.

*M. chimaera* is able to form biofilms and may persist in water samples [8], which may partially explain its long-lasting occurrence in water-containing HCUs used for open-chest cardiac surgery [4,6]. While device contamination during the production process [14] and a subsequent air-borne transmission [13,30,31] have been proposed as transmission pathways for this recent outbreak, the clinical significance of our findings in ECMO devices and the potential risks for patients remain to be elucidated. However, several characteristics seen in our study differ from those observed in connection with cardiac surgery. First, *M. chimaera* was detected in water from two different providers of thermoregulatory devices, thus rendering contamination during the production process of a single, specific device relatively unlikely. Second, an air-borne transmission of *M. chimaera* from the ECMO device to the patient could not be demonstrated. The ECMO-related thermoregulatory devices are, in contrast to HCUs used during cardiac surgery, air-tight and closed systems. In line with this, we did not find any evidence of detectable mycobacteria upon air sampling in patient rooms during ECMO treatment.

It is important to note that the mere diagnosis of an atypical mycobacterium in a bronchial specimen is not necessarily linked to an ongoing infection [32]. Indeed, following careful retrospective patient chart assessment in our study, we consider the detection of *M. chimaera* in bronchial aspirates from three patients during or after ECMO treatment not to be evidently associated with the *M. chimaera* contamination of the thermoregulatory devices. Patient 2 of the aforementioned patients, who was highly immunocompromised, suffered from pulmonary graft vs. host disease after allogenic SCT and had not been tested for atypical mycobacteria before ECMO therapy. Thus, it cannot be excluded that he might have already been colonised with atypical mycobacteria before ECMO treatment. In contrast, Patients 1 and 3 had been negative in mycobacterial sputum analyses before ECMO initiation. However, both patients had also been exposed to HCUs during open-chest surgery. Further molecular diagnostics could have helped to further characterise the origin of the *M. chimaera* strains found in these patients, e.g. through molecular analyses comparing their genetic characteristics to those of *M. chimaera* strains detected in water from ECMO devices and HCUs used in cardiac surgery.

ECMO is a life-saving technology, in particular for patients with severe respiratory failure despite maximal medical treatment [16,33,34]. Such patients suffer from comorbidities, are frequently immunocompromised and thus a highly vulnerable population. We therefore recommend that specific investigations for *M. chimaera* should be carried out in more ECMO centres to identify whether this pathogen constitutes a potentially relevant infectious agent in ECMO treatment settings. In our study, we were unable to identify a distinct source of the *M. chimaera* contamination. No mycobacteria were found in the tap water used to fill the thermoregulatory devices, thus rendering a contamination with environmental mycobacteria unlikely. A contamination during the manufacturing process of the thermoregulatory devices cannot be excluded, but seems rather unlikely because devices of different manufacturers were affected. Additionally, cross-contamination from cardiac HCUs used in the operating theatre might also have occurred, e.g. when surgery was performed on ECMO patients and the same oxygenator was used on different thermoregulatory devices.

Our study has several limitations. First, it is a single-centre study with a limited sample size. Yet, our report is the first systematic assessment of *M. chimaera* beyond the setting of cardiac surgery, and therefore provides important additional evidence. Second, our clinical patient analysis is retrospective, mainly due to the fact that we initiated the current study only after the publication of the first outbreak reports related to HCU devices used during cardiac surgery. The retrospective design of our patient chart review might have biased some of our results, specifically pertaining to repeated sampling procedures for *M. chimaera*. Future research on this topic should thus preferably employ a prospective study design. Third, repeated sampling of water from thermoregulatory devices might have further improved the detection rate and more sophisticated microbiological analyses e.g. whole-genome sequencing and comparison of *M. chimaera* isolates obtained from water and patient samples could have elucidated the genetic relatedness of the mycobacterial strains. Fourth, additional microbiological investigations of all water samples pertaining to e.g. *Legionella* spp. and *Pseudomonas* spp. might have helped to better assess the water quality and to better quantify the contamination of the thermoregulatory devices.

## Conclusions

Patients receiving ECMO treatment are often highly immunocompromised and prone to opportunistic infections, including those caused by atypical mycobacteria. The detection of *M. chimaera* in a considerable amount of water samples from thermoregulatory ECMO devices in our centre should encourage further research in other hospital centres to elucidate the origin of such contamination. Additionally, the hitherto unclear clinical relevance of *M. chimaera* in the setting of ECMO treatment needs to be assessed. Strict adherence to disinfection protocols published by the manufacturers of thermoregulatory devices as well as continued microbiological surveillance for *M. chimaera* are recommended to minimise the risk of infection.
